# Utilization and Outcomes of Structural Heart Disease Interventions in Patients With Prior Mediastinal Radiation

**DOI:** 10.1016/j.jacadv.2025.102319

**Published:** 2025-11-08

**Authors:** Mahmoud Ismayl, Hasaan Ahmed, Andrew M. Goldsweig, Joerg Herrmann, Mackram F. Eleid, Charanjit S. Rihal, Mayra Guerrero

**Affiliations:** a Department of Cardiovascular Medicine, Mayo Clinic, Rochester, Minnesota, USA; b Department of Cardiovascular Medicine, Baystate Medical Center, Springfield, Massachusetts, USA; c Department of Cardiovascular Medicine, University of Massachusetts-Baystate, Springfield, Massachusetts, USA

**Keywords:** cancer, LAAO, MTEER, radiation, TAVR, TMVR

## Abstract

**Background:**

Outcomes of structural heart disease (SHD) interventions may be affected by prior mediastinal radiation.

**Objectives:**

The objective of the study was to evaluate the outcomes of SHD interventions among patients with vs without prior mediastinal radiation.

**Methods:**

We analyzed the Nationwide Readmissions Database (2016-2022) to identify patients aged ≥18 years with prior mediastinal radiation who underwent SHD interventions. The primary outcome was in-hospital mortality. Secondary outcomes included procedural complications, resource utilization (length of stay, costs, discharge disposition), and 90-day readmissions. In-hospital outcomes were analyzed with logistic regression and readmissions with Cox proportional hazards models.

**Results:**

Among 810,849 weighted hospitalizations for transcatheter aortic valve replacement, mitral transcatheter edge-to-edge repair, transcatheter mitral valve replacement, and left atrial appendage occlusion, 1.3% included patients with prior mediastinal radiation. Utilization rates (procedures/100,000 hospitalizations) were higher in patients with vs without prior mediastinal radiation for transcatheter aortic valve replacement (577 vs 254), mitral transcatheter edge-to-edge repair (70 vs 35), and transcatheter mitral valve replacement (11 vs 4) (all *P* < 0.001). There were no significant differences in adjusted in-hospital mortality, complications, or resource utilization between patients with vs without prior mediastinal radiation following any of the 4 SHD interventions. Ninety-day readmissions were similar after transcatheter aortic and mitral valve interventions but higher after left atrial appendage occlusion (adjusted HR: 1.51; 95% CI: 1.21-1.89) in patients with vs without prior mediastinal radiation.

**Conclusions:**

Transcatheter aortic and mitral valve interventions are performed more frequently in patients with vs without prior mediastinal radiation with similar in-hospital outcomes and readmissions. Readmissions after LAAO are higher in patients with vs without prior mediastinal radiation.

Mediastinal radiation, often used alongside chemotherapy and surgery, is a cornerstone in the management of various chest malignancies, improving survival in appropriately selected patients.^1^, ^2^, ^3^ However, mediastinal radiation also causes long-term adverse effects to the heart and surrounding structures through direct toxicity and progressive tissue fibrosis.^3^^,^^4^ Consequently, patients treated with mediastinal radiation are at risk of developing cardiovascular complications including valvular disease, arrhythmias, and accelerated atherosclerosis.^3^^,^^4^ Radiation-induced cardiac damage has become the leading cause of noncancer-related mortality in this patient population.^3^^,^^4^ Managing cardiovascular complications in these patients is particularly challenging due to the heightened surgical risks associated with prior radiation therapy that result from chest wall scarring, pulmonary fibrosis, and delayed wound healing due to stem cell death.^1^^,^^5^^,^^6^ These factors increase perioperative morbidity and mortality, with prior studies reporting adverse outcomes among patients with prior mediastinal radiation undergoing surgical valvular interventions.^1^^,^^5^^,^^6^

Transcatheter structural heart disease (SHD) interventions have emerged as promising alternatives to surgical interventions, especially for patients at a high surgical risk. However, limited data have been reported regarding the impact of prior mediastinal radiation on outcomes of SHD interventions, including transcatheter aortic valve replacement (TAVR), mitral transcatheter edge-to-edge repair (MTEER), transcatheter mitral valve replacement (TMVR), and left atrial appendage occlusion (LAAO). Therefore, we analyzed the Nationwide Readmissions Database (NRD) to evaluate trends and outcomes of SHD interventions in patients with vs without prior mediastinal radiation.

## Methods

### Data source and ethics statement

Hospitalization data were abstracted from the NRD, which is a part of the Healthcare Cost and Utilization Project (HCUP) family of databases sponsored by the Agency for Healthcare Research and Quality.^7^ The specific data supporting this study's findings are available from the corresponding author on request. The NRD is the largest publicly available, fully deidentified, all-payer inpatient health care readmission database in the United States. The NRD covers approximately 18 million unweighted hospitalizations each year with a diverse patient population, growing from 27 states in 2016 to 30 states in 2022 (Supplemental Table 1).^7^ When weighted, the NRD extrapolates to the national level, representing approximately 35 million hospitalizations each year. The unweighted sample represents approximately 50% of all U.S. hospitalizations. Up to 40 discharge diagnoses and 25 procedure codes are collected for each patient using International Classification of Diseases-10th Revision (ICD-10) codes.^8^ For readmission analyses, the NRD provides a validated patient linkage variable (“NRD_VisitLink”) that allows tracking of readmissions within a single calendar year.^7^ The design and methodology of the NRD have been described previously.^9^, ^10^, ^11^ This study followed the Strengthening the Reporting of Observational Studies in Epidemiology (STROBE) reporting guideline (Supplemental Table 2)^12^ and was exempt from the requirements of the Mayo Clinic Institutional Review Board because the NRD is a fully deidentified, Health Insurance Portability and Accountability Act-compliant database that is publicly available from the HCUP website (www.hcup-us.ahrq.gov).

### Study population and patient selection

**Figure 1:**
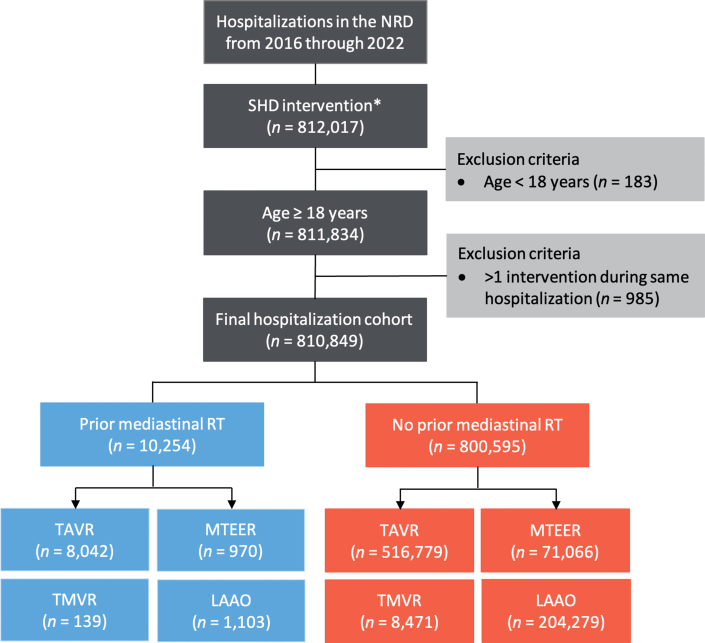
**Study Flow Diagram Showing Inclusion and Exclusion Criteria** Hospitalization counts represent national-level estimates. ∗Includes TAVR, MTEER, TMVR, or LAAO. LAAO = left atrial appendage occlusion; MTEER = mitral transcatheter edge-to-edge repair; NRD = Nationwide Readmissions Database; RT = radiation therapy; SHD = structural heart disease; TAVR = transcatheter aortic valve replacement; TMVR = transcatheter mitral valve replacement.

We analyzed the NRD from January 2016 through December 2022 to identify hospitalizations in which adult patients (age ≥18 years) underwent TAVR, MTEER, TMVR, or percutaneous LAAO using ICD-10 procedure codes (02RF37H, 02RF38H, 02RF3JH, 02RF3KH, X2RF332, 02RF37Z, 02RF38Z, 02RF3JZ, 02RF3KZ for TAVR; 02UG3JH, 02UG3JZ, 02QG3ZE, 02QG3ZZ for MTEER; 02RG37H, 02RG38H, 02RG3JH, 02RG3KH, 02RG37Z, 02RG38Z, 02RG3JZ, 02RG3KZ for TMVR; and 02L73DK for percutaneous LAAO). We excluded hospitalizations in which the patient was aged <18 years. Hospitalizations with >1 SHD intervention during the same admission were excluded from the outcomes analysis but not from the utilization and trend analyses (Figure 1). For hospitalizations that met the inclusion criteria, we stratified the patients into 2 cohorts based on the presence or absence of prior mediastinal radiation. Similar to previous administrative claims database studies, this study identified prior mediastinal radiation by the presence of ICD-10 diagnostic codes for prior radiation as well as ICD-10 diagnostic codes for history of breast cancer, lung cancer, Hodgkin lymphoma, or other mediastinal tumors.^1^ These ICD-10 codes have previously been validated to identify patients with those cancer conditions treated with prior radiation.^13^, ^14^, ^15^ The use of administrative claims databases for identifying patients with these types of cancers has been previously reported to achieve a specificity of 95% to 99% and sensitivity of 83% to 93%.^13^^,^^16^, ^17^, ^18^

A complete list of ICD-10 diagnosis and procedure codes used in this study is presented in Supplemental Table 3.

When evaluating 90-day readmissions, we excluded hospitalizations in which the patient died during the index hospitalization as well as any discharge that occurred after September 30 of each calendar year because the NRD follows patients within a single calendar year and does not capture readmissions across calendar years. Therefore, to enable a full 90-day follow-up for all discharges, we only used data from January 1 to September 30 of each year for the analysis on 90-day readmissions. In patients who had multiple 90-day readmissions, only the first readmission was included in the analysis. The NRD does not provide data on out-of-hospital deaths, and therefore patients who died at home within 90 days would be counted as patients without a readmission within 90 days.

### Study outcomes

Temporal trends and procedure utilization rates, defined as the number of procedures performed per 100,000 hospitalizations, were reported. The primary outcome was in-hospital all-cause mortality. Secondary outcomes included in-hospital complications: stroke, acute kidney injury, major bleeding, need for blood transfusion, vascular complications (defined as a composite of arteriovenous fistula, aneurysm, hematoma, retroperitoneal bleeding, and venous thromboembolism), permanent pacemaker placement, and cardiac tamponade, as well as hospital length of stay (LOS), total hospital costs (inflation adjusted to 2022 U.S. dollars),^19^ and discharge disposition. Charge-to-cost ratio files were used to convert charges to costs at the individual hospital level. Readmissions within 90 days were also evaluated.

### Statistical analysis

Descriptive statistics were presented as percentages for categorical variables and as medians with IQRs for continuous variables. Categorical variables were compared using the Pearson chi-square test or Fisher exact test as appropriate. Continuous variables were compared using the Mann-Whitney *U* test.

Trend analyses from 2016 through 2022 were conducted using linear regression models incorporating an autoregressive correlation structure. Multivariable logistic regression was used to compare in-hospital outcomes of SHD interventions between patients with vs without prior mediastinal radiation. The logistic regression model included the following variables: age, sex, primary payer, median household income quartile by zip code, hospital location (urban/rural) and teaching status, number of hospital beds, admission type (elective/nonelective) and day (weekend/weekday), Elixhauser and Charlson Comorbidity Index scores, and relevant comorbidities (Supplemental Table 4). Adjustment variables were selected a priori based on their clinical significance and on their likely influence on in-hospital outcomes. The results from these models are presented as adjusted ORs (aORs) with 95% CIs.

The probability of 90-day readmission, stratified on the basis of prior mediastinal radiation, was graphically displayed using the Kaplan-Meier method and was compared using the log-rank test. To estimate the adjusted HR (aHR) for 90-day readmissions for patients with vs without prior mediastinal radiation, an a priori multivariable Cox proportional hazards regression analysis was performed using the same variables listed previously (Supplemental Table 4). Estimated aHRs were reported with corresponding 95% CIs. The assumptions of Cox proportional hazards regression were graphically assessed using log-log plots and tested based on Schoenfeld residuals. Visual inspection of log-log survival plots for all covariates indicated that the curves were approximately parallel over time, suggesting that the proportional hazards assumption was satisfied. Formal testing using Schoenfeld residuals showed no significant correlation between residuals and time (all *P* > 0.05), indicating that the proportional hazards assumption was appropriate.

Complete data were available for all variables except primary payer (missing 0.1%), median household income quartile by zip code (missing 1.1%), and type of admission (missing 0.1%). As the overall missing values were minimal (<1.5%) and limited to only 3 variables, they were assumed to be missing at random, and the level of bias was likely small. Missing values were handled with listwise deletion and were not included in the regression analysis.

For all statistical analyses, a 2-tailed *P* < 0.05 was considered statistically significant. Given the large sample size, not all statistically significant *P* values are clinically significant and therefore require careful interpretation. All statistical analyses were performed using Stata (version 17, StataCorp) software, accounting for the complex NRD sampling design, including clustering at the hospital level (“HOSP_NRD”), which accounts for correlation of admissions within the same hospital but not within the same patient, stratification (“NRD_STRATUM”), and discharge weights (“DISCWT”) to estimate national-level effects per HCUP-NRD recommendations similar to prior studies.^7^^,^^20^, ^21^, ^22^, ^23^

## Results

### Utilization of SHD interventions

A total of 810,849 weighted hospitalizations for SHD interventions were identified for analysis (Figure 1), of which, 1.3% included patients with prior mediastinal radiation. Patients with prior mediastinal radiation constituted 1.5%, 1.4%, 1.6%, and 0.5% of all patients who underwent TAVR, MTEER, TMVR, and LAAO, respectively. Patients with prior mediastinal radiation were significantly more likely to undergo TAVR (577 vs 254 procedures/100,000 hospitalizations), MTEER (70 vs 35), and TMVR (11 vs 4), but less likely to undergo LAAO (79 vs 100) than patients without prior mediastinal radiation (all *P* < 0.001) (Central Illustration).

**Figure 2:**
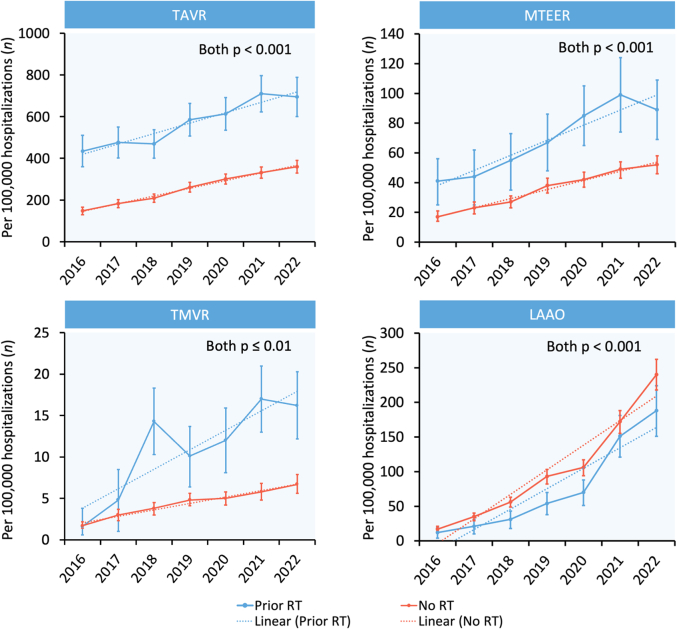
**Trends in Structural Heart Disease Interventions Among Patients With Prior Mediastinal Radiation** Error bars represent 95% CIs. Dotted lines represent linear trends. Abbreviations as in Figure 1.

**Figure 3:**
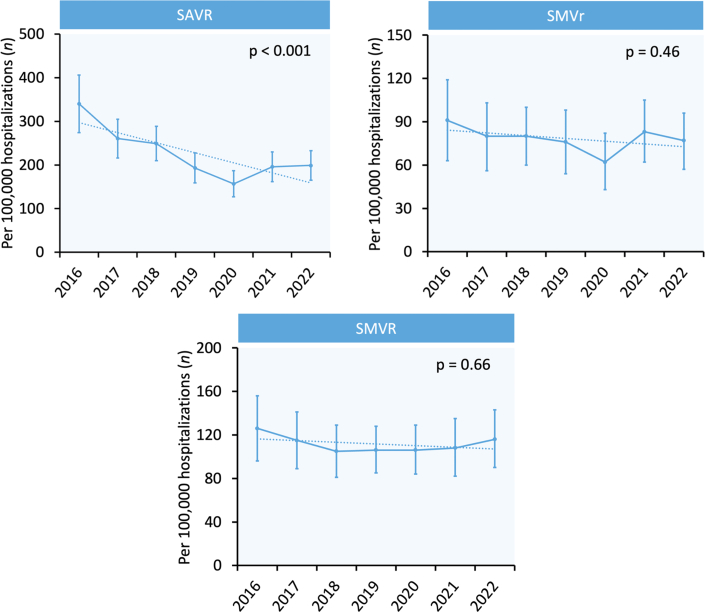
**Trends in Surgical Valve Interventions Among Prior Mediastinal Radiation Patients** Error bars represent 95% CIs. Dotted lines represent linear trends. SAVR = surgical aortic valve replacement; SMVr = surgical mitral valve repair; SMVR = surgical mitral valve replacement.

**Figure 4:**
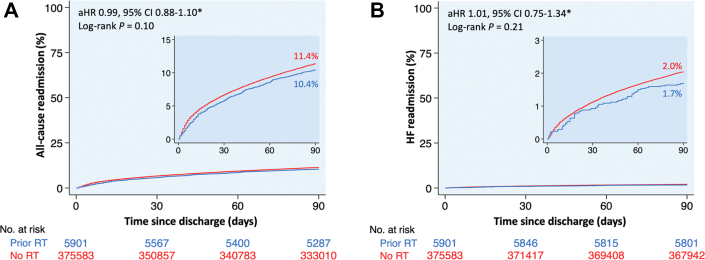
**Kaplan-Meier Curves of 90-Day Readmissions Following Transcatheter Aortic Valve Replacement** Kaplan-Meier curves of (A) all-cause and (B) HF readmissions at 90 days following TAVR, stratified by the presence or absence of prior mediastinal radiation. ∗Multivariable regression model adjusted for age, sex, primary payer, median income quartile, hospital location (urban/rural) and teaching status, number of hospital beds, admission type (elective/nonelective) and day (weekend/weekday), Elixhauser and Charlson Comorbidity Index scores, and relevant comorbidities (Supplemental Table 4). aHR = adjusted HR; HF = heart failure; other abbreviations as in Figure 1.

**Figure 5:**
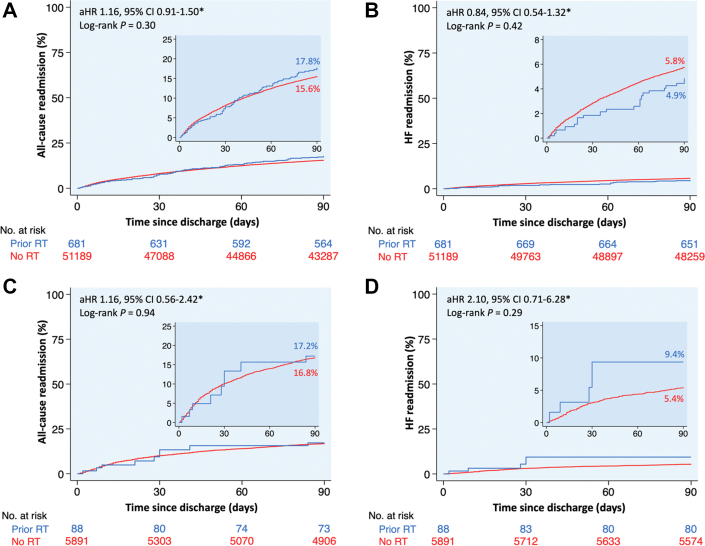
**Kaplan-Meier Curves of 90-Day Readmissions Following Mitral Transcatheter Edge-to-Edge Repair and Transcatheter Mitral Valve Replacement** Kaplan-Meier curves of all-cause and HF readmissions at 90 days following MTEER (A: all-cause, B: HF) and TMVR (C: all-cause, D: HF), stratified by the presence or absence of prior mediastinal radiation. ∗Multivariable regression model adjusted for age, sex, primary payer, median income quartile, hospital location (urban/rural) and teaching status, number of hospital beds, admission type (elective/nonelective) and day (weekend/weekday), Elixhauser and Charlson Comorbidity Index scores, and relevant comorbidities (Supplemental Table 4). Abbreviations as in Figures 1 and 4.

**Figure 6:**
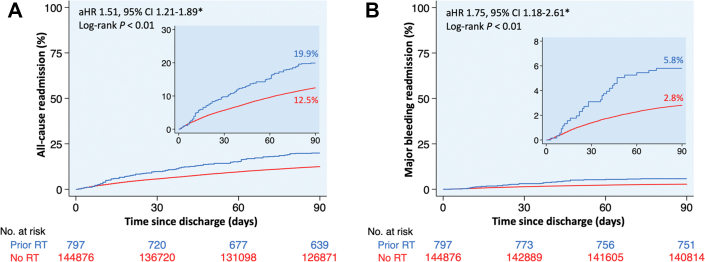
**Kaplan-Meier Curves of 90-Day Readmissions Following Left Atrial Appendage Occlusion** Kaplan-Meier curves of (A) all-cause and (B) major bleeding readmissions at 90 days following LAAO, stratified by the presence or absence of prior mediastinal radiation. ∗Multivariable regression model adjusted for age, sex, primary payer, median income quartile, hospital location (urban/rural) and teaching status, number of hospital beds, admission type (elective/nonelective) and day (weekend/weekday), Elixhauser and Charlson Comorbidity Index scores, and relevant comorbidities (Supplemental Table 4). Abbreviations as in Figures 1 and 4.

**Central Illustration:**
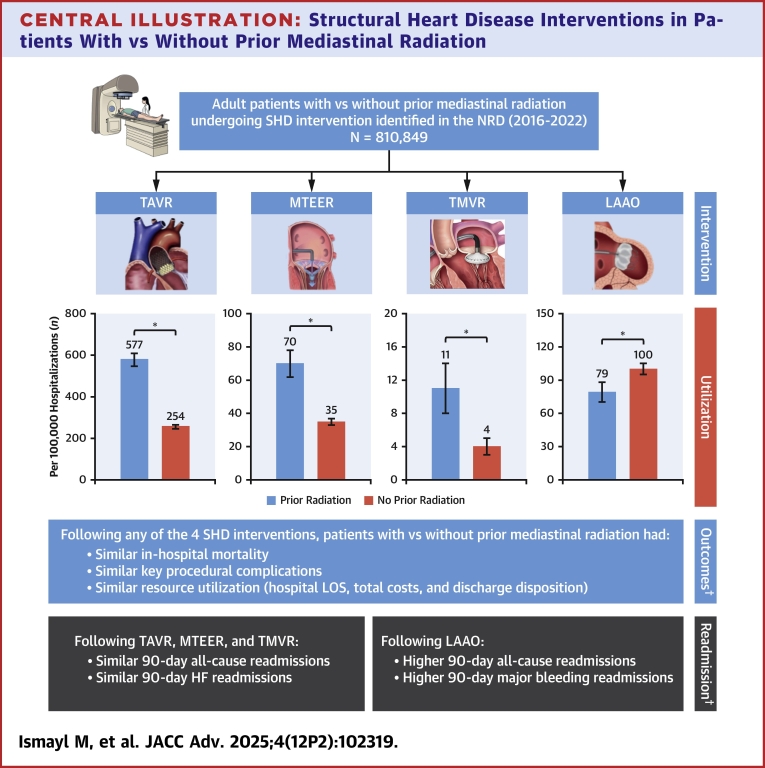
**Structural Heart Disease Interventions in Patients With vs Without Prior Mediastinal Radiation** Error bars represent 95% CIs. ∗*P* < 0.001. ^†^Multivariable regression model adjusted for age, sex, primary payer, median income quartile, hospital location (urban/rural) and teaching status, number of hospital beds, admission type (elective/nonelective) and day (weekend/weekday), Elixhauser and Charlson Comorbidity Index scores, and relevant comorbidities (Supplemental Table 4). HF = heart failure; LAAO = left atrial appendage occlusion; LOS = length of stay; MTEER = mitral transcatheter edge-to-edge repair; NRD = Nationwide Readmissions Database; SHD = structural heart disease; TAVR = transcatheter aortic valve replacement; TMVR = transcatheter mitral valve replacement.

In multivariable regression analysis, prior mediastinal radiation was independently associated with higher odds of TAVR (aOR: 1.78; 95% CI: 1.70-1.86), MTEER (aOR: 1.36; 95% CI: 1.24-1.49), and TMVR (aOR: 1.39; 95% CI: 1.07-1.82), and lower odds of LAAO (aOR: 0.57; 95% CI: 0.52-0.63) compared with no prior mediastinal radiation.

### Temporal trends in SHD interventions

From 2016 through 2022, the number of SHD interventions per 100,000 hospitalizations increased significantly in patients both with and without prior mediastinal radiation for all 4 procedures: TAVR (435 to 695 [prior radiation] and 148 to 360 [no prior radiation]), MTEER (41 to 89 [prior radiation] and 17 to 52 [no prior radiation]), TMVR (2 to 16 [prior radiation] and 2 to 7 [no prior radiation]), and LAAO (12 to 188 [prior radiation] and 17 to 240 [no prior radiation]) (all *p*_trend_ ≤ 0.01). In contrast, the number of surgical valve interventions per 100,000 hospitalizations in patients with prior mediastinal radiation decreased for surgical aortic valve replacement (AVR) (340 to 199, *P*_trend_ < 0.001) and remained similar for surgical mitral valve repair (91 to 77, *P*_trend_ = 0.46) and replacement (126 to 116, *P*_trend_ = 0.66) from 2016 through 2022. Annual trends for transcatheter SHD interventions and surgical valve interventions in patients with prior mediastinal radiation are shown in Figures 2 and 3, respectively.

### Patient and hospital characteristics

There were notable differences in demographic, hospital, and clinical characteristics between patients with vs without prior mediastinal radiation undergoing SHD interventions. Patients with prior mediastinal radiation undergoing valvular interventions were more likely to be female (79.9% [7,308/9,151] vs 43.3% [258,231/596,316], *P* < 0.001) and have chronic pulmonary disease (32.3% [2,956/9,151] vs 25.8% [153,826/596,316], *P* < 0.001) compared to those without prior mediastinal radiation. Patients with prior mediastinal radiation undergoing TAVR and MTEER had lower Elixhauser and Charlson Comorbidity Index scores than those without prior mediastinal radiation, driven by the lower rates of diabetes mellitus (28.7% [2,589/9,012] vs 36.6% [214,995/587,845]), hypertension (84.4% [7,608/9,012] vs 89.2% [524,299/587,845]), coronary artery disease (61.8% [5,574/9,012] vs 67.9% [399,359/587,845]), renal failure (23.7% [2,133/9,012] vs 34.3% [201,819/587,845]), obstructive sleep apnea (11.9% [1,072/9,012] vs 15.4% [90,638/587,845]), prior myocardial infraction (10.0% [904/9,012] vs 11.9% [69,786/587,845]), and prior coronary artery bypass grafting (8.0% [725/9,012] vs 14.7% [86,640/587,845]) (all *P* < 0.001). Baseline characteristics, stratified by the presence or absence of prior mediastinal radiation, are shown in Table 1.

**Table 1:** Baseline Characteristics Stratified by Presence vs Absence of Prior Mediastinal Radiation

	TAVR			MTEER			TMVR			LAAO		
	Prior RT (n = 8,042)	No RT (n = 516,779)	*P* Value	Prior RT (n = 970)	No RT (n = 71,066)	P Value	Prior RT (n = 139)	No RT (n = 8,471)	*P* Value	Prior RT (n = 1,103)	No RT (n = 204,279)	*P* Value
Demographic characteristics												
Age (years)	77 (70-83)	80 (74-85)	<0.001	77 (70-83)	78 (70-84)	0.03	72 (66-79)	75 (67-81)	0.21	77 (73-82)	77 (71-82)	0.14
18-64	13.3	5.9	<0.001	14.1	15.4	0.02	23.4	20.6	0.33	5.0	6.6	0.09
65-74	26.2	22.6		26.8	21.5		35.3	27.8		30.6	32.3	
75-84	40.5	43.5		39.7	39.5		33.0	39.3		51.8	46.8	
85+	20.0	28.0		19.4	23.6		8.3	12.3		12.6	14.3	
Biological sex												
Male	20.6	57.0	<0.001	16.0	55.9	<0.001	22.2	44.8	<0.001	18.2	58.8	<0.001
Female	79.4	43.0		84.0	44.1		77.8	55.2		81.8	41.2	
Primary payer												
Medicare	84.2	89.2	<0.001	85.5	82.7	0.29	78.1	81.8	0.13	93.1	89.9	0.04
Medicaid	1.2	1.4		1.5	3.3		<7.9^a^	5.1		<1.0^a^	1.1	
Private insurance	12.8	6.9		10.6	11.7		18.4	10.9		5.1	7.2	
Self-pay	0.4	0.3		<1.1^a^	0.8		<7.9^a^	0.6		<1.0^a^	0.3	
Other	1.4	2.2		1.8	1.5		0	1.6		<1.0^a^	1.5	
Income quartile^b^												
I	17.9	20.9	<0.001	17.0	22.4	0.001	23.4	22.2	0.82	18.5	22.1	0.05
II	23.9	27.9		23.7	25.3		26.6	26.1		26.2	27.8	
III	27.1	27.0		26.1	26.7		23.2	27.6		28.2	27.6	
IV	31.1	24.2		33.2	25.6		26.8	24.1		27.1	22.5	
Hospital characteristics												
Location/teaching status												
Metropolitan nonteaching	7.8	10.0	<0.001	3.6	8.2	0.004	<7.9^a^	6.0	0.76	10.4	11.5	0.10
Metropolitan teaching	91.7	88.8		95.6	90.9		94.6	93.1		88.9	86.6	
Nonmetropolitan hospital	0.5	1.2		<1.1^a^	0.9		0	0.9		<1.0^a^	1.9	
Bed size^c^												
Small	5.7	6.0	0.67	2.5	4.7	0.09	<7.9^a^	3.8	0.59	7.4	7.1	0.80
Medium	21.6	22.1		22.1	20.9		17.5	15.6		21.8	23.1	
Large	72.7	71.9		75.4	74.4		80.8	80.6		70.8	69.8	
Elective admission	86.6	83.6	<0.001	80.5	79.6	0.66	83.8	68.8	0.005	95.7	94.0	0.07
Weekend admission	2.4	3.3	0.002	3.6	3.8	0.84	<7.9^a^	7.2	0.58	1.1	0.5	0.13
Clinical characteristics												
Elixhauser Comorbidity Index	5 (4-7)	6 (4-7)	<0.001	5 (4-7)	6 (4-7)	<0.001	6 (5-8)	6 (5-8)	0.69	4 (3-6)	4 (3-5)	0.09
Charlson Comorbidity Index	2 (1-4)	3 (1-4)	<0.001	2 (1-4)	3 (1-4)	0.03	3 (2-4)	3 (2-4)	0.89	2 (1-3)	2 (1-3)	0.19
0	7.4	7.3	<0.001	8.0	7.1	0.01	9.4	3.7	0.11	17.1	22.8	0.05
1	21.4	19.5		26.5	21.1		18.1	20.4		24.4	24.1	
2	22.2	20.3		18.2	18.3		23.9	19.3		23.2	18.8	
≥3	49.0	52.9		47.3	53.5		48.6	56.6		35.3	34.3	
Diabetes mellitus	30.0	38.0	<0.001	18.2	26.2	<0.001	26.0	29.1	0.52	30.6	34.2	0.07
Hypertension	85.0	90.0	<0.001	79.6	83.3	0.04	86.8	83.4	0.46	89.1	87.0	0.15
Dyslipidemia	73.8	75.3	0.03	58.4	63.4	0.02	68.8	62.9	0.31	65.5	64.0	0.47
Nicotine/tobacco use	41.1	39.6	0.07	37.2	37.4	0.92	39.6	36.3	0.57	47.2	38.2	<0.001
Alcohol abuse	0.6	1.5	<0.001	<1.1^a^	1.7	0.31	<7.9^a^	1.7	0.54	1.3	1.4	0.75
Drug abuse	0.4	0.6	0.21	<1.1^a^	1.2	0.02	0	1.8	0.26	0	0.3	0.24
Obesity	20.0	21.8	0.008	9.9	12.8	0.07	12.0	15.0	0.45	18.2	18.5	0.86
Coronary artery disease	63.7	69.0	<0.001	46.5	60.2	<0.001	55.7	59.6	0.52	41.2	47.9	0.002
Peripheral vascular disease	20.8	21.2	0.57	24.0	24.2	0.90	14.2	18.7	0.34	14.0	15.6	0.32
Congestive heart failure	71.8	72.8	0.18	83.3	85.0	0.23	83.5	89.3	0.18	39.6	38.7	0.68
Renal failure	23.2	33.8	<0.001	27.6	38.2	<0.001	24.7	39.7	0.006	22.3	24.0	0.36
Dialysis dependent	1.2	2.8	<0.001	2.0	2.8	0.26	<7.9^a^	3.2	0.38	1.3	2.2	0.23
Liver disease	2.9	3.8	0.009	1.2	4.2	<0.001	15.8	6.9	0.01	3.7	3.0	0.37
Chronic pulmonary disease	32.4	25.8	<0.001	31.3	25.1	0.003	33.6	31.4	0.68	32.2	21.2	<0.001
Obstructive sleep apnea	12.2	15.6	<0.001	9.4	14.1	0.003	8.7	15.9	0.06	17.2	19.5	0.23
Coagulopathy	8.1	10.8	<0.001	10.4	10.8	0.81	21.2	22.1	0.87	4.4	4.0	0.69
Malnutrition	1.2	1.6	0.05	1.5	2.4	0.15	<7.9^a^	4.2	0.43	0	0.2	0.34
Dementia	2.2	3.9	<0.001	2.8	2.7	0.93	<7.9^a^	2.0	0.43	2.5	2.7	0.73
Depression	9.9	8.6	0.004	11.6	8.2	0.008	13.7	10.1	0.40	11.9	7.8	0.001
Previous history												
Myocardial infarction	9.9	11.4	0.007	11.1	15.3	0.008	<7.9^a^	10.9	0.14	12.1	11.9	0.89
Stroke/TIA	11.3	11.5	0.74	11.9	10.2	0.26	11.9	13.6	0.65	22.8	21.5	0.45
Cardiac arrest	0.6	0.5	0.11	<1.1^a^	1.1	0.83	0	0.8	0.44	<1.0^a^	0.5	0.46
PCI	20.3	21.1	0.19	14.4	18.5	0.02	9.0	10.6	0.66	16.6	16.5	0.99
CABG	7.9	14.4	<0.001	9.2	17.2	<0.001	21.6	24.8	0.54	7.6	12.0	0.002
ICD	2.3	2.2	0.94	10.8	12.4	0.28	<7.9^a^	8.0	0.35	3.4	5.7	0.03
PPM	8.1	8.7	0.16	7.4	10.0	0.05	24.1	14.0	0.007	15.5	16.7	0.50

Data presented as median (IQR) or %. Two authors (M.I. and H.A.) independently verified the International Classification of Diseases-10th Revision (ICD-10) codes that corresponded to each comorbidity (Supplemental Table 3), and any disagreements in inclusion or exclusion of *ICD-10* codes were discussed with a third author (A.M.G).

CABG = coronary artery bypass grafting; ICD = implantable cardioverter-defibrillator; LAAO = left atrial appendage occlusion; MTEER = mitral transcatheter edge-to-edge repair; PCI = percutaneous coronary intervention; PPM = permanent pacemaker; RT = radiation therapy; TAVR = transcatheter aortic valve replacement; TIA = transient ischemic attack; TMVR = transcatheter mitral valve replacement.

a Cell counts <11 are not reportable per HCUP guidelines.

b Estimated median household incomes are ZIP code–specific, updated annually, and classified into 4 quartiles indicating the poorest to wealthiest populations.

c Bed-size categories are based on inpatient beds and are specific to the hospital’s location and teaching status. A more detailed explanation of all the variables in the NRD, including the specific dollar amounts in each category of median household income and the number of hospital beds in each category, is available online (https://hcup-us.ahrq.gov/db/nation/nrd/nrddde.jsp).

### In-hospital outcomes, los, and costs of SHD interventions

Despite the higher utilization rates of SHD interventions in patients with prior mediastinal radiation, no significant differences in in-hospital outcomes were observed after adjustment for potential confounders. With TAVR, there were no statistically significant differences in in-hospital mortality (aOR: 0.63; 95% CI: 0.37-1.07) or key post-TAVR complications between patients with vs without prior mediastinal radiation after adjustment for potential confounders. Similarly, in-hospital mortality following MTEER (aOR: 0.37; 95% CI: 0.09-1.47), TMVR (aOR: 1.55; 95% CI: 0.72-3.36), and LAAO (aOR: 2.85; 95% CI: 0.93-4.53) and key complications were similar between patients with vs without prior mediastinal radiation after adjustment for potential confounders.

In addition, there were no significant differences in hospital LOS, total costs, and discharge disposition between patients with vs without prior mediastinal radiation following any of the 4 SHD interventions. In-hospital outcomes, LOS, and costs of SHD interventions, stratified by the presence or absence of prior mediastinal radiation, are presented in Table 2.

**Table 2:** In-Hospital Outcomes of SHD Interventions Stratified by Presence vs Absence of Prior Mediastinal Radiation

	TAVR			MTEER			TMVR			LAAO		
	Prior RT (n = 8,042)	No RT (n = 516,779)	*P* Value	Prior RT (n = 970)	No RT (n = 71,066)	*P* Value	Prior RT (n = 139)	No RT (n = 8,471)	*P* Value	Prior RT (n = 1,103)	No RT (n = 204,279)	*P* Value
Complications												
Death												
%	0.6	1.3	0.008	<1.1^a^	1.8	0.10	11.3	5.4	0.07	<1.0^a^	0.1	0.02
uOR (95% CI)	0.50 (0.30-0.84)	Ref.	-	0.36 (0.10-1.28)	Ref.	-	2.25 (0.91-5.56)	Ref.	-	4.17 (1.18-14.68)	Ref.	-
aOR (95% CI)^b^	0.63 (0.37-1.07)	Ref.	-	0.37 (0.09-1.47)	Ref.	-	1.55 (0.72-3.36)	Ref.	-	2.85 (0.93-4.53)	Ref.	-
Stroke												
%	1.8	2.1	0.10	<1.1^a^	1.2	0.22	<7.9^a^	2.2	0.92	<1.0^a^	0.7	0.41
uOR (95% CI)	0.82 (0.65-1.04)	Ref.	-	0.54 (0.20-1.47)	Ref.	-	1.08 (0.25-4.65)	Ref.		0.62 (0.19-1.96)	Ref.	-
aOR (95% CI)^b^	0.82 (0.65-1.05)	Ref.	-	0.55 (0.20-1.49)	Ref.	-	1.76 (0.39-8.02)	Ref.		0.62 (0.19-2.00)	Ref.	-
Acute kidney injury												
%	4.9	9.2	<0.001	8.4	14.5	<0.001	24.6	22.8	0.73	1.8	1.9	0.85
uOR (95% CI)	0.51 (0.44-0.60)	Ref.	-	0.54 (0.39-0.75)	Ref.	-	1.10 (0.63-1.95)	Ref.	-	0.95 (0.55-1.63)	Ref.	-
aOR (95% CI)^b^	0.90 (0.77-1.05)	Ref.	-	0.57 (0.40-0.81)	Ref.	-	1.42 (0.79-2.56)	Ref.	-	0.92 (0.51-1.64)	Ref.	-
Major bleeding												
%	1.0	1.0	0.99	<1.1^a^	0.7	0.79	<7.9^a^	1.5	0.82	<1.0^a^	0.2	0.63
uOR (95% CI)	1.00 (0.74-1.36)	Ref.	-	1.13 (0.47-2.72)	Ref.	-	0.79 (0.11-5.88)	Ref.	-	1.42 (0.34-6.01)	Ref.	-
aOR (95% CI)^b^	0.96 (0.70-1.30)	Ref.	-	1.03 (0.41-2.58)	Ref.	-	0.67 (0.07-5.96)	Ref.	-	1.35 (0.32-5.74)	Ref.	-
Blood transfusion												
%	4.4	4.7	0.36	3.7	4.8	0.24	17.0	11.3	0.23	1.7	1.3	0.38
uOR (95% CI)	0.93 (0.80-1.08)	Ref.	-	0.76 (0.48-1.20)	Ref.	-	1.61 (0.73-3.55)	Ref.	-	1.33 (0.70-2.53)	Ref.	-
aOR (95% CI)^b^	0.93 (0.80-1.08)	Ref.	-	0.70 (0.44-1.11)	Ref.	-	1.41 (0.63-3.17)	Ref.	-	1.25 (0.65-2.40)	Ref.	-
Vascular complications												
%	3.4	3.7	0.30	1.9	2.9	0.22	<7.9^a^	5.6	0.53	1.6	0.9	0.10
uOR (95% CI)	0.91 (0.77-1.09)	Ref.	-	0.66 (0.33-1.29)	Ref.	-	0.70 (0.23-2.11)	Ref.	-	1.70 (0.90-3.20)	Ref.	-
aOR (95% CI)^b^	0.90 (0.75-1.08)	Ref.	-	0.67 (0.33-1.34)	Ref.	-	0.85 (0.28-2.62)	Ref.	-	1.47 (0.79-2.76)	Ref.	-
PPM placement												
%	6.1	6.6	0.19	<1.1^a^	0.5	0.25	0	1.9	0.28	<1.0^a^	0.2	0.60
uOR (95% CI)	0.92 (0.81-1.04)	Ref.	-	1.67 (0.69-4.08)	Ref.	-	-	Ref.	-	0.59 (0.08-4.20)	Ref.	-
aOR (95% CI)^b^	1.08 (0.95-1.23)	Ref.	-	1.68 (0.69-4.12)	Ref.	-	-	Ref.	-	0.58 (0.08-4.12)	Ref.	-
Cardiac tamponade												
%	0.5	0.7	0.18	<1.1^a^	0.5	0.58	7.9	0.8	<0.001	<1.0^a^	0.4	0.57
uOR (95% CI)	0.73 (0.45-1.17)	Ref.	-	1.33 (0.49-3.67)	Ref.	-	9.84 (2.74-35.32)	Ref.	-	0.67 (0.16-2.76)	Ref.	-
aOR (95% CI)^b^	0.64 (0.39-1.03)	Ref.	-	0.98 (0.36-2.70)	Ref.	-	3.26 (0.78-13.58)	Ref.	-	0.53 (0.13-2.19)	Ref.	-
Resource utilization												
LOS (days)	2 (1-3)	2 (1-4)	0.10	2 (1-3)	2 (1-4)	0.12	4 (1-7)	4 (1-10)	0.09	1 (1-1)	1 (1-1)	0.15
Hospital cost ($)	51,097 (36,139-75,971)	53,202 (37,295-83,165)	0.12	47,117 (31,713-71,707)	48,602 (33,603-81,547)	0.19	79,990 (67,517-238,317)	66,541 (43,858-109,910)	0.07	29,764 (21,755-37,090)	28,924 (20,771-42,529)	0.77
Discharge disposition												
Routine	75.1	73.1	0.09	78.7	75.9	0.26	72.0	60.1	0.12	95.1	94.6	0.78
Transfer to short-term hospital	0.2	0.2		<1.1^a^	0.3		<7.9^a^	0.5		0	0.1	
Transfer to SNF or ICF	5.5	8.4		4.4	6.5		<7.9^a^	10.6		1.2	1.8	
Home health care	19.2	18.3		16.6	17.3		19.6	28.8		3.7	3.5	

Data presented as %, OR (95% CI), or median (IQR). The International Classification of Diseases-10th Revision (ICD-10) codes corresponding to each of the in-hospital outcomes were identified with the same process used to identify comorbidity codes (Supplemental Table 3).

aOR = adjusted OR; ICF = intermediate care facility; LOS = length of stay; SNF = skilled nursing facility; uOR = unadjusted OR; other abbreviations as in Table 1.

a Cell counts <11 are not reportable per HCUP guidelines.

b Multivariable regression model adjusted for age, sex, primary payer, median income quartile, hospital location (urban/rural) and teaching status, number of hospital beds, admission type (elective/nonelective) and day (weekend/weekday), Elixhauser and Charlson Comorbidity Index scores, and relevant comorbidities (Supplemental Table 4).

### 90-day readmission

After adjustment for potential confounders using multivariable regression analysis, patients with prior mediastinal radiation had similar all-cause and heart failure readmissions at 90 days following TAVR, MTEER, and TMVR compared to those without prior mediastinal radiation (Figures 4 and 5). Among patients who underwent LAAO, 90-day all-cause readmissions were higher in patients with vs without prior mediastinal radiation (aHR: 1.51; 95% CI: 1.21-1.89), primarily driven by more readmissions for major bleeding (aHR: 1.75; 95% CI: 1.18-2.61) (Figure 6).

## Discussion

This analysis of patients undergoing SHD interventions using the large, nationally representative NRD yielded several novel findings (Central Illustration): 1) transcatheter aortic and mitral valve interventions are performed more frequently in patients with vs without prior mediastinal radiation; 2) from 2016 through 2022, the use of transcatheter SHD interventions in patients with prior mediastinal radiation increased, whereas surgical AVR decreased and mitral valve surgery remained similar; 3) adjusted in-hospital mortality, key complications, and resource utilization of common SHD interventions were comparable between patients with vs without prior mediastinal radiation; 4) following transcatheter aortic and mitral valve interventions, 90-day all-cause and heart failure readmissions were similar between patients with vs without prior mediastinal radiation; 5) however, following LAAO, all-cause and major bleeding readmissions were higher among patients with vs without prior mediastinal radiation.

### Utilization and temporal trends in SHD interventions

Our study found that transcatheter aortic and mitral valve interventions were performed more frequently among patients with prior mediastinal radiation compared to those without. This finding likely relates to the higher rates of valvular heart disease associated with mediastinal radiation resulting in more interventions.^4^

Prior studies evaluating trends in SHD interventions have noted an increasing use across various patient populations.^24^, ^25^, ^26^, ^27^, ^28^ The present study demonstrates a significant increase in transcatheter aortic and mitral valve interventions in patients with prior mediastinal radiation over the study period, whereas surgical AVR decreased and mitral valve surgery remained similar. These trends likely reflect the challenges posed by mediastinal radiation, including fibrosis, calcification, and structural damage to cardiac and mediastinal tissues, which increase the surgical risk, making transcatheter interventions preferred.^1^^,^^29^ Common complications in these patients, such as porcelain aorta, constrictive pericarditis, reduced ejection fraction, and poor pulmonary function heighten perioperative morbidity and mortality.^1^^,^^29^ Transcatheter interventions may circumvent many of these challenges by avoiding sternotomy and cardiopulmonary bypass, providing safer and less invasive alternatives to surgical interventions.^1^^,^^29^ In addition, technological advancements in procedural techniques, increased operator experience, enhanced safety outcomes of transcatheter therapies, expanded patient eligibility criteria, and the widespread availability of both SHD interventions and advanced imaging modalities across institutions likely increased their use in patients with prior mediastinal radiation who otherwise are poor candidates for surgery.^1^^,^^29^^,^^30^

In addition to valvular heart disease, patients with prior mediastinal radiation are at an increased risk of developing atrial fibrillation requiring anticoagulation, likely due to radiation-induced atrial remodeling, myocardial injury, and fibrosis.^31^^,^^32^ They are also at an increased risk of bleeding due to thrombocytopenia from oncologic treatments such as chemotherapy and immunotherapy, as well as radiation-induced coagulopathy and gastric injury.^33^, ^34^, ^35^, ^36^ Given their high bleeding risk and poor surgical candidacy—due to mediastinal adhesions and impaired wound healing^1^^,^^5^^,^^6^—surgical left atrial appendage closure techniques, such as the AtriClip, are often not feasible, making percutaneous LAAO a more suitable alternative. However, despite these considerations, patients with prior mediastinal radiation were less likely to undergo LAAO compared to those without in the present study.

### In-hospital outcomes, los, and costs of SHD interventions

Prior studies have demonstrated adverse outcomes in patients with prior mediastinal radiation undergoing surgical valvular interventions.^1^^,^^5^^,^^6^ In addition, radiation-induced atrial remodeling may contribute to an increased risk of complications during LAAO, including perforation and pericardial effusion, as well as postprocedure complications, including impaired endothelialization resulting in device-related thrombus and systemic thromboembolism.

Adjusted in-hospital mortality and key complications of common SHD interventions were comparable between patients with vs without prior mediastinal radiation in our study. This reassuring finding of similar in-hospital safety outcomes following SHD interventions is congruent with prior studies^5^^,^^29^ and emphasizes that SHD interventions are as safe in patients with prior mediastinal radiation as in the general population.

There were no significant differences in hospital LOS, total costs, or discharge disposition between patients with vs without prior mediastinal radiation following any of the 4 SHD interventions. These findings parallel the similar rates of in-hospital complications between patients with vs without prior mediastinal radiation, as prior studies have associated in-hospital procedural complications during LAAO with significantly prolonged hospital stays and higher hospitalization costs.^37^^,^^38^

### 90-day readmission

Following transcatheter aortic and mitral valve interventions, our study found similar all-cause and heart failure readmissions at 90 days between patients with vs without prior mediastinal radiation, which aligns with prior studies.^5^^,^^29^ Agarwal et al^5^ reported similar all-cause and heart failure readmissions at 30 and 180 days among patients with vs without prior mediastinal radiation who underwent TAVR. Similarly, Elbadawi et al^29^ reported similar 30-day readmission rates following MTEER between patients with vs without prior mediastinal radiation. These findings reflect advancements in transcatheter valvular techniques, which have improved procedural safety and mitigated challenges associated with mediastinal fibrosis from prior radiation.^39^ The comparable readmission risk despite the complexities of cancer and radiation-related comorbidities, bleeding risks, and the dynamics of ongoing oncologic treatment underscores the safety and efficacy of transcatheter aortic and mitral valve interventions in this high-risk population with a safety profile similar to that of the general population.

Our study found that 90-day all-cause and major bleeding readmissions following LAAO were higher in patients with prior mediastinal radiation. A prior study by Zhang et al^40^ reported that the most common cause for readmission following LAAO in cancer patients was gastrointestinal bleeding. Patients referred for LAAO frequently have prior bleeding or intolerance to oral anticoagulation therapy, and prior mediastinal radiation may further increase bleeding risk. Potential factors contributing to the higher observed rate of major bleeding—and the associated increase in all-cause readmissions—in patients with prior mediastinal radiation following LAAO include: 1) radiation-induced coagulopathy, which can alter hemostasis and increase bleeding tendency;^34^ 2) radiation-induced gastrointestinal mucosal injury, particularly in the gastric fundus or cardia, resulting in ulceration and gastrointestinal bleeding;^35^^,^^36^ 3) treatment-related thrombocytopenia from chemotherapy, immunotherapy, or radiation; 4) lower body mass index, sarcopenia, and frailty associated with cancer treatments, which are independent risk factors for bleeding;^41^^,^^42^ and 5) heterogeneity in post-LAAO antithrombotic regimens and duration, which could have influenced bleeding outcomes. Further prospective studies are warranted to better quantify bleeding risk and define optimal antithrombotic strategies after LAAO in patients with prior mediastinal radiation.

### Limitations and strengths

Our study has several important limitations. First, in a retrospective NRD study using administrative claims codes, incorrect coding could lead to inaccurate data. Second, the retrospective nature of the study makes it subject to inherent selection bias. Third, detailed baseline and procedural characteristics such as cancer treatment history, timing of SHD interventions relative to cancer diagnosis and treatment, echocardiographic and computed tomographic data, study devices, access sites, and periprocedural medications including antithrombotic regimen were unavailable, which may introduce unmeasured bias. Fourth, the NRD does not provide detailed information on pre-existing bioprosthetic valves or annuloplasty rings in the aortic and mitral positions, limiting our ability to accurately determine the proportion of TAVR and TMVR procedures that involved valve-in-prosthetic valve or valve-in-ring interventions. Although the ICD-10 code Z95.2 is available to indicate the presence of a prosthetic heart valve, it is infrequently and inconsistently used due to several limitations. Specifically, it does not specify the anatomical position of the valve (aortic, mitral, tricuspid, or pulmonic), the type of valve (bioprosthetic or mechanical), or the procedural approach (surgical or transcatheter). Furthermore, it does not capture the presence of mitral valve rings. Nevertheless, given the absence of Food and Drug Administration-approved transcatheter therapies for mitral valve-in-native valve replacement during the study period, it is likely that most TMVR cases in our cohort represented valve-in-prosthetic valve or valve-in-ring procedures. This is supported by the relatively low mortality rates observed in our study compared to those reported in the largest retrospective series of TMVR for valve-in-mitral annular calcification (25.0% and 53.7% at 30-days and 1-year, respectively), where outcomes are known to be significantly worse.^43^ Fifth, validated risk scores such as the Society of Thoracic Surgeons score are not captured by the NRD, limiting patient risk assessment. Sixth, because the NRD is a discharge-level database, patients may contribute multiple hospitalizations; outside of readmission analyses using the patient linkage variable, repeat admissions for the same individual cannot be tracked, representing an inherent limitation of the NRD. Nevertheless, repeat admissions for the same patient undergoing the same procedure are exceedingly rare and unlikely to meaningfully impact our results. Seventh, given the lack of data on out-of-hospital deaths, patients who died at home within 90 days were counted as patients without a readmission within 90 days. Finally, because the NRD does not allow longitudinal tracking of patients beyond a single calendar year, we were unable to report a median and IQR of follow-up time; instead, follow-up was limited to a maximum of 90 days after discharge. Studies exploring the long-term outcomes of SHD interventions in patients with prior mediastinal radiation are still needed.

Despite these limitations, the present study adds meaningfully to the literature by describing the relative utilization and comparative outcomes of SHD interventions in patients with vs without prior mediastinal radiation after adjustment for demographic and clinical characteristics. The NRD is well validated for outcomes studies like this one and undergoes serial data accuracy checks and quality control. In addition, the NRD population is geographically diverse, providing a nationally representative sample of centers and operators, and hence reliably reflects real-world practice and outcomes across the United States.

## Conclusions

Transcatheter aortic and mitral valve interventions are performed more frequently in patients with prior mediastinal radiation compared to those without, with comparable in-hospital safety outcomes and 90-day all-cause and heart failure readmissions. In contrast, LAAO is performed less frequently in patients with prior mediastinal radiation and is associated with a higher risk of 90-day all-cause and major bleeding readmissions in this population.

Perspectives **COMPETENCY IN MEDICAL KNOWLEDGE:** Among patients with prior mediastinal radiation, the use of transcatheter SHD interventions increased from 2016 through 2022, whereas surgical AVR decreased and mitral valve surgery remained relatively stable. Compared to patients without prior mediastinal radiation, those with prior radiation more frequently underwent transcatheter aortic and mitral valve procedures, with similar in-hospital outcomes and 90-day all-cause and heart failure readmissions. In contrast, LAAO was performed less frequently in patients with prior mediastinal radiation and was associated with higher 90-day all-cause and major bleeding readmissions in this population.

**TRANSLATIONAL OUTLOOK:** Further studies are warranted to assess the long-term outcomes of SHD interventions in patients with prior mediastinal radiation and to investigate the underlying factors contributing to increased bleeding risk following LAAO in this high-risk population.

## Funding support and author disclosures

Dr Goldsweig reports consulting for Philips and Conformal Medical and speaking for Philips. Dr Guerrero has received institutional research grant support from 10.13039/100006520Edwards Lifesciences. All other authors have reported that they have no relationships relevant to the contents of this paper to disclose.
